# A randomized, double-blind, placebo-controlled phase 2 study evaluating the efficacy and safety of romiplostim treatment of patients with low or intermediate-1 risk myelodysplastic syndrome receiving lenalidomide

**DOI:** 10.1186/1756-8722-5-71

**Published:** 2012-11-29

**Authors:** Eunice S Wang, Roger M Lyons, Richard A Larson, Sunil Gandhi, Delong Liu, Carmen Matei, Bart Scott, Kuolung Hu, Allen S Yang

**Affiliations:** 1 Leukemia Service, Department of Medicine, Roswell Park Cancer Institute, Elm and Carlton Streets, Buffalo, NY 14263, USA; 2 Cancer Care Centers of South Texas/US Oncology, 4411 Medical Drive, Suite 100, San Antonio, TX, 78229, USA; 3 Comprehensive Cancer Center, University of Chicago, 5841 S. Maryland Avenue, MC-2115, Chicago, Illinois, 60637, USA; 4 Cancer and Blood Disease Center, 421 N. Lecanto Highway, Lecanto, Florida, 34461, USA; 5 Westchester Medical Center, Munger Pavilion 250, Valhalla, New York, 10595, USA; 6 Rocky Mountain Cancer Centers/US Oncology, 3027 North Circle Drive, Colorado Springs, CO, 80909, USA; 7 Fred Hutchinson Cancer Research Center, 1100 Fairview Ave. N, D1-100, Seattle, Washington, 98109, USA; 8 Amgen Inc., One Amgen Center Drive, Thousand Oaks, California, 91320, USA

**Keywords:** Romiplostim, Lenalidomide, Thrombocytopenia, Myelodysplastic syndrome

## Abstract

**Background:**

Lenalidomide treatment in myelodysplastic syndrome (MDS) may lead to thrombocytopenia and dose reductions/delays. This study evaluated the safety and tolerability of the thrombopoietin mimetic romiplostim and its effects on the incidence of clinically significant thrombocytopenic events (CSTEs) in lower risk MDS patients receiving lenalidomide.

**Methods:**

Patients were assigned to weekly placebo (n = 12) or romiplostim 500 μg (n = 14) or 750 μg (n = 13) for four 28-day lenalidomide cycles.

**Results:**

The treatment groups were generally similar with respect to baseline disease characteristics. Del(5q) abnormalities were noted in 1 (8%) patient in the placebo group, 3 (21%) in the romiplostim 500 μg group, and two (15%) in the 750 μg group. CSTEs were noted in 8 (67%) patients in the placebo group, 4 (29%) in the romiplostim 500 μg group, and 8 (62%) in the romiplostim 750 μg group. Throughout the study, median platelet counts trended lower in placebo-treated than in romiplostim-treated patients. Thrombocytopenia-related adjustments in lenalidomide occurred in 6 (50%) patients in the placebo group, 5 (36%) in the romiplostim 500 μg group, and 2 (15%) in the 750 μg group. Although the percentages of patients who received platelet transfusions were similar across treatment groups, there was a trend toward lower numbers of transfusions in both romiplostim groups during each treatment cycle. There were two serious treatment-related adverse events during the treatment period (cerebrovascular accident, placebo; worsening thrombocytopenia, romiplostim 500 μg). Two patients (romiplostim 500 and 750 μg, respectively) had an increase in bone marrow blasts to >20% during treatment, but had no post-treatment biopsy to confirm or exclude the diagnosis of progression to AML.

**Conclusions:**

These data suggest that romiplostim administered to MDS patients during lenalidomide treatment may decrease the frequency of dose reductions/delays due to thrombocytopenia. Additional study is needed to confirm the results of this preliminary trial.

**Trial registration:**

ClinicalTrials.gov NCT00418665

## Introduction

Myelodysplastic syndromes (MDS) encompass a heterogeneous group of malignancies of hematopoietic progenitor cells. MDS is characterized by stem-cell-derived clonal myelopoiesis, hypercellular bone marrow with dysplastic changes, ineffective hematopoiesis, and increased apoptosis resulting in peripheral cytopenias [1-3]. MDS is one of the most common hematologic malignancies in older individuals [1], with an annual incidence between 75 and 162 per 100,000 persons 65 years or older [4,5]. Patients often present with complications related to anemia (fatigue), neutropenia (infections), and/or thrombocytopenia (bleeding). Over the course of the disease, thrombocytopenia occurs in 40–65% of patients with MDS and can result in serious hemorrhagic complications, leading to death in 14–24% of patients [6]. Worsening thrombocytopenia is closely linked to the underlying MDS disease biology and has been associated with higher risk of transformation to acute myeloid leukemia (AML) and lower overall survival [7,8]. Currently, platelet transfusion and aminocaproic acid are the only supportive treatments for thrombocytopenia in MDS patients.

The pathogenesis of MDS is incompletely understood but involves genetic, epigenetic, and immune-mediated mechanisms [9]. Chromosomal aberrations are found in half of patients at diagnosis [9]. Interstitial deletion of the long arm of chromosome 5 (del[5q]) is the most frequently reported cytogenetic aberration in MDS and has been reported in up to 15% of cases [10,11]. Lenalidomide [12], an immunomodulatory agent, is approved for the treatment of patients with MDS associated with del(5q) on the basis of results of prior studies demonstrating that this agent resulted in transfusion independence in 67% of treated patients [13]. However, treatment-related thrombocytopenia occurs in 44–74% of lenalidomide-treated MDS patients [13,14]. Because this myelosuppression is dose dependent, cytopenias constitute the most common reason for lenalidomide dose adjustments, which have been reported in up to 84% of treated MDS patients [13,15]. In a study of the relationship between lenalidomide-related cytopenias and treatment response, thrombocytopenia was predictive of red blood cell transfusion independence (TI) in lower risk MDS patients with del(5q) [16]. This finding suggests that response to lenalidomide depends on effective suppression of the MDS clone [16]. Given that thrombocytopenia is common in MDS patients responding to lenalidomide and reflects the pharmacological activity of the drug, concomitant treatment with a thrombopoietic agent to reduce clinically significant bleeding events related to low platelet counts may be of significant benefit to affected MDS patients.

Romiplostim is a Fc-peptide fusion protein (peptibody) that increases platelet production by binding to and activating the thrombopoietin receptor initiating megakaryopoiesis [17]. It has no sequence homology to thrombopoietin. Romiplostim is indicated for the treatment of thrombocytopenia in patients with chronic immune thrombocytopenia (ITP) who have had an insufficient response to corticosteroids, immunoglobulins, or splenectomy [18]. It has been investigated for treatment of MDS patients with or without del (5q) [19-23]. In phase 1/2 studies, 46–65% of lower-risk thrombocytopenic MDS patients receiving romiplostim achieved an International Working Group (IWG)-defined platelet response [19,20,24]. In other studies, administration of romiplostim in combination with methyltransferase inhibitors appeared to confer therapeutic benefits in patients with low- or intermediate 1/2-risk (per IPSS score) MDS [23,25].

We conducted this study to evaluate the safety and tolerability of romiplostim and its effects on the incidence of clinically significant thrombocytopenic events (CSTEs) in patients with low or intermediate-1-risk (“lower-risk”) MDS receiving lenalidomide therapy.

## Methods

### Study design and ethical considerations

This phase 2, multicenter, randomized, double-blind, placebo-controlled study was conducted at 24 centers throughout the United States from March 2007 to March 2009. The protocol was reviewed and approved by the appropriate institutional review board at each center before any patients were recruited. The trial was conducted in accordance with the principles of the Food and Drug Administration (FDA) and International Conference on Harmonisation (ICH) Good Clinical Practice (GCP) regulations/guidelines. This trial was registered at http://www.clinicaltrials.gov as #NCT00418665.

The study consisted of a double-blind treatment period, during which patients received four 28-day cycles of lenalidomide plus weekly injections of placebo or romiplostim, and an optional open-label extension period, during which patients could receive lenalidomide plus romiplostim. Patients returned to the center weekly during the treatment period, and again 1 day after the end of the fourth lenalidomide cycle for a follow-up visit. Patients who completed the 16-week treatment period continued lenalidomide and discontinued romiplostim for at least 4 weeks prior to returning for an end-of-treatment visit at week 20. After this visit, patients could continue lenalidomide and were eligible to receive romiplostim in the optional open-label extension period. All patients who entered the extension period had an end-of-study visit 4 weeks after the last dose of romiplostim. After the extension phase, patients could choose to enter a separate optional second open-label extension study [21]. Written, informed consent was obtained from all patients or a legally acceptable representative before any study-specific procedures were performed.

### Patients

Adult patients were eligible to participate in the study if they had a diagnosis of MDS based on World Health Organization (WHO) 2001 classification of marrow findings [26] with IPSS lower-risk MDS disease [27], an Eastern Cooperative Oncology (ECOG) performance status of 0–2, and adequate liver and kidney function. All patients agreed to receive ≥ 4 cycles of lenalidomide capsules 10 mg by mouth daily. Patients were excluded if they had previous exposure to > 3 cycles of lenalidomide or exposure to lenalidomide within the last 30 days, or if they had a history of leukemia or aplastic anemia, stem cell transplantation, or prior malignancy (other than *in situ* cervical cancer or basal cell cancer of the skin) unless treated with curative intent and without evidence of disease for ≥3 years before randomization. Patients who had active or uncontrolled infections, uncontrolled cardiovascular disease, or a history of arterial or venous thrombosis within the past year were also excluded, as were patients who had received IL-11 within 4 weeks of screening, any investigational drug or device < 4 weeks previously, or any other thrombopoietic growth factor.

### Randomization and treatment

Patients were assigned identification numbers from an interactive voice response system (IVRS) and randomly assigned in a 1:1:1 ratio to receive placebo or romiplostim 500 μg or 750 μg. Patients were stratified by baseline platelet count (≥ 50 × 10^9^/L or < 50 × 10^9^/L). During the treatment period, all patients received a 10-mg lenalidomide capsule orally each day for four 28-day cycles, for a planned total dose of 1120 mg; doses were reduced or delayed when necessary as directed in the product labeling [12]. In addition, patients received subcutaneous injections of placebo or romiplostim 500 μg or 750 μg each week for 16 weeks. If a patient had a platelet count > 450 × 10^9^/L, investigational product was withheld until the platelet count fell to < 200 × 10^9^/L. Once the platelet count fell to < 200 × 10^9^/L, investigational product was resumed on the next scheduled dosing day. Patients whose dose of lenalidomide was delayed continued to receive their weekly doses of romiplostim. Patients who were thrombocytopenic for ≥ 4 weeks after discontinuation of romiplostim could resume romiplostim treatment whether or not they were receiving lenalidomide.

During the open-label extension, patients who had received romiplostim during the treatment period remained on the same dose, and patients who had received placebo began treatment with romiplostim 500 μg. All patients continued lenalidomide 10 mg daily. If a patient discontinued lenalidomide, romiplostim was also discontinued temporarily. Patients who became thrombocytopenic (as evidenced by an average of at least two platelet counts ≤ 50 × 10^9^/L with one count on the day romiplostim was restarted) at least 4 weeks after the last dose of romiplostim and lenalidomide could remain on study and restart romiplostim at a dose of 750 μg weekly until the end of the extension period.

During the double-blind portion of the study, investigational product was packaged in two identical vials for each scheduled dose for each patient. Patients received 1.5 mL of investigational product in each dose—1 mL from one vial and 0.5 mL from the second vial. Patients in the 500 μg group received 1 mL of romiplostim and 0.5 mL of placebo, patients in the 750 μg group received 1.5 mL of romiplostim, and patients in the placebo group received 1.5 mL of placebo.

Throughout the study, investigators were allowed to prescribe any concomitant medications or treatments deemed necessary to provide adequate supportive care except for the following: any medication known or suspected to affect platelet production, immunomodulatory agents, histone deacetylase inhibitors, cyclosporine, mycophenolate, any myelosuppressive chemotherapy other than lenalidomide, and any other investigational product. Rescue medication, defined as any medication, including platelet transfusions, administered to raise platelet counts, was given only when a patient was at immediate risk.

### Assessments

Throughout the treatment and extension periods, patients returned to the study center weekly for administration of investigational product and review of adverse events, bleeding events, and concomitant medications. During the treatment period, samples were drawn weekly for complete blood counts (including platelet counts and differential) and blood chemistry analyses. Blood samples were also intermittently collected for tests for antibodies to romiplostim. Bone marrow biopsy and aspirate samples were obtained for assessment of bone marrow reticulin or collagen formation and cytogenetics at the screening and end-of-treatment visits. Progression to AML was determined on the basis of a marrow or peripheral blast cell count ≥ 20% with confirmation 4 weeks after withdrawal of romiplostim. During the extension period, samples were drawn weekly for platelet counts, every 2 weeks for complete blood counts and differential, and every 4 weeks for blood chemistry analyses. Blood samples were collected for tests for antibodies to romiplostim at weeks 1, 17, and 33 and at the end-of-study visit, and for cytogenetics at the end-of-study visit. Bone marrow biopsy and aspirate samples were also collected at the end-of-study visit.

### Data analysis

The proposed sample size was 12 patients per group. CSTEs were defined as (a) any platelet count obtained from week 3 of cycle 1 through the follow-up visit that was < 50 × 10^9^/L and/or (b) the receipt of a platelet transfusion at any time through the follow-up visit. The distance from the observed difference to the sides of the 95% confidence intervals (95% CIs) was 0.362, with the assumption that the rates of CSTEs would be 20% for romiplostim and 50% for placebo.

No formal hypothesis testing was planned for this dose-finding study. Descriptive statistics for demographic and baseline characteristics, safety, and efficacy were summarized for all patients. For categorical variables, the number and percentage of patients in each category were summarized. Continuous variables were summarized by number, mean, standard deviation (SD), median, Q1 (25th percentile), Q3 (75th percentile), and minimum and maximum values. For efficacy variables, 95% exact binomial confidence intervals for the incidence were provided for each treatment group and for the difference between treatment groups. The Mantel-Haenszel common odds ratios controlling for baseline platelet counts were estimated along with 95% confidence intervals. Additional summary by del(5q) status was also provided.

Efficacy analyses were performed using the set of all randomized patients. Patients who discontinued the study before the occurrence of an event were considered not to have had the event and were included in the analysis unless otherwise noted. The primary efficacy endpoint was the percentage of patients who had a CSTE. Additional efficacy endpoints included the percentage of patients who had a reduction or delay in lenalidomide dose due to thrombocytopenia during the treatment period; the percentage of patients who received ≥ 1 platelet transfusions and the total number of units administered during the treatment period; the percentage of patients who had a complete response (CR), partial response (PR), or overall response (CR + PR) at the end of the treatment period based on the 2006 modified IWG guideline [28]; and the incidence of bleeding events. The exposure-adjusted bleeding event rates were provided with 95% CIs. Post hoc endpoints included the proportion of patients whose platelet counts were < 20 × 10^9^/L at any point during the treatment cycle or overall, by baseline platelet count and for all patients, and the percent decreases in platelet counts from day 1 of the treatment cycle to the nadir for each treatment cycle.

Safety analyses were performed using the set of patients who received at least one dose of investigational product. Patients were analyzed according to the treatment actually received. Safety was assessed on the basis of the incidence of adverse events, anti-romiplostim antibody formation, and formation of antibodies that cross-react with endogenous thrombopoietin (eTPO).

## Results

### Patients

Of the 39 patients randomized, 12 were assigned to receive weekly placebo, 14 to receive weekly romiplostim 500 μg, and 13 to receive weekly romiplostim 750 μg. The median (range) age of the patients was 74 (39–90) years, and 62% were male (Table 1). The treatment groups were generally similar with respect to baseline disease characteristics (Table 1). Six patients (15%) had MDS characterized by del(5q) abnormalities: one in the placebo group, three in the romiplostim 500 μg group, and two in the 750 μg group. Two patients, one each from the placebo and romiplostim 500 μg groups, were deemed ineligible for the study because they had an IPSS score > 1.0 (“higher-risk”). Although these patients did not receive any investigational product, they were included in the analyses of efficacy. Two patients randomized to the placebo group each erroneously received one dose of romiplostim during the treatment period. These patients are included in the placebo group of the efficacy analysis set and in the romiplostim group of the safety analysis set.

**Table 1:** Demographic characteristics

**Baseline demographics, n (%)**	**Placebo**	**Romiplostim**		**Total**
		**500 μg**	**750 μg**	
	**(N = 12)**	**(N = 14)**	**(N = 13)**	**(N = 39)**
Sex – Male, n (%)	8 (67)	8 (57)	8 (62)	24 (62)
Race – White or Caucasian, n (%)	11 (92)	13 (93)	12 (92)	36 (92)
Age (years), median (range)	79 (39–87)	75 (49–90)	65 (49–83)	74 (39–90)
Platelets (× 10^9^/L), n (%)				
<50 × 10^9^/L	5 (42)	5 (36)	5 (39)	15 (39)
≥50 × 10^9^/L	6 (50)	8 (57)	8 (62)	22 (56)
Unknown^*^	1 (8)	1 (7)	0 (0)	2 (5)
IPSS score, n (%)				
0	4 (33)	4 (29)	6 (46)	14 (36)
0.5	3 (25)	6 (43)	4 (31)	13 (33)
1.0	3 (25)	2 (14)	3 (23)	8 (21)
1.5^**†**^	1 (8)	1 (7)	0 (0)	2 (5)
Unknown^*^	1 (8)	1 (7)	0 (0)	2 (5)
MDS duration (y), median (range)	2.6 (0–9)	0.5 (0–6)	0.5 (0–7)	0.6 (0–9)
MDS diagnosis - n (%)				
RA	1 (8)	0 (0)	0 (0)	1 (3)
RARS	2 (17)	2 (14)	2 (15)	6 (15)
RAEB-1	2 (17)	3 (21)	3 (23)	8 (21)
RCMD	4 (33)	4 (29)	4 (31)	12 (31)
RCMD-RS	0 (0)	0 (0)	1 (8)	1 (3)
MDS-U	1 (8)	1 (7)	1 (8)	3 (8)
MDS associated with isolated del(5q)	1 (8)	3 (21)	2 (15)	6 (15)
Unknown^*^	1 (8)	1 (7)	0 (0)	2 (5)
Cytogenetic findings^‡^				
Normal/diploid	8 (67)	7 (50)	10 (77)	25 (64)
+8	1 (8)	2 (14)	1 (8)	4 (10)
-Y	2 (17)	1 (7)	0	3 (8)
del(5q)	1 (8)	3 (21)	2 (15)	6 (15)
del(7q)	0	1 (7)	0	1 (3)
del(12p)	1 (8)	1 (7)	0	2 (5)
Other	1 (8)	0	0	1 (3)
Complex (>3 abnormalities)	0	0	0	0
Unknown	1 (8)	1 (7)	0	2 (5)

Legend*: del 5q* deletion 5q cytogenetic abnormality, *IPSS* International Prognostic Scoring System, *MDS* myelodysplastic syndrome, *MDS-U* myelodysplastic syndrome, unclassified, *RA* refractory anemia, *RAEB* refractory anemia with excess blasts, *RARS* refractory anemia with ringed sideroblasts, *RCMD* refractory cytopenia with multilineage dysplasia, *RCMD-RS* refractory cytopenia with multilineage dysplasia and ringed sideroblasts.

^*^Information not available for two ineligible patients.

^†^Patients permitted to enter study despite protocol violation of IPSS score greater 1.0 (protocol violation).

^‡^Patients could have more than one abnormality (and so could be counted more in more than one row).

**Figure 1:**
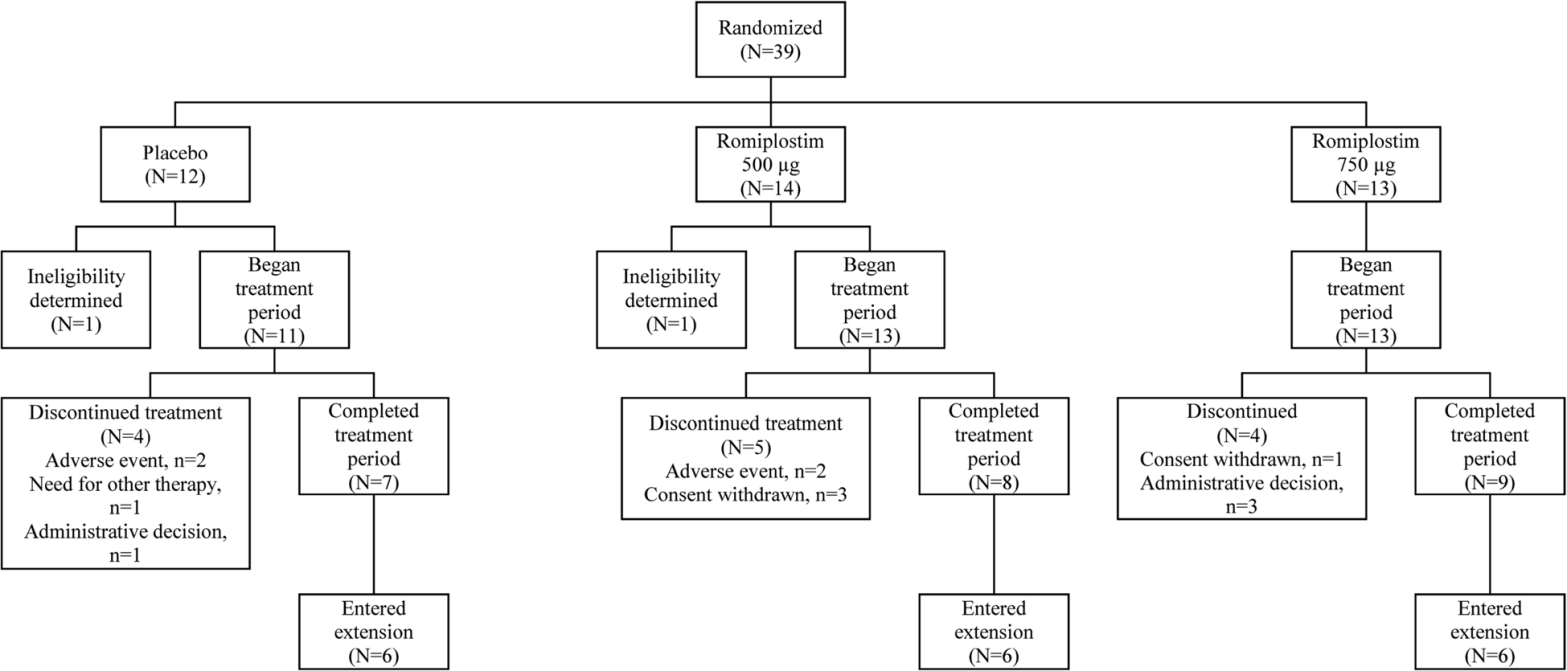
Patient disposition.

A total of 24 patients completed the treatment period, and 13 (33%) discontinued (Figure 1). The most common reasons for discontinuation were adverse event, withdrawal of consent, and administrative decisions, each occurring in four (10%) patients. Eighteen (46%) patients entered the optional extension period, and 12 (31%) discontinued during the extension period. The baseline characteristics of the patients who entered the extension period were similar to those of the patients who entered the initial treatment period. During the extension period, the most common reasons for discontinuation were requirement for alternative therapy in 4 (10%) patients and administrative decisions in 5 (13%). One patient in the romiplostim 750 μg group died during the extension period from intestinal obstruction, which was not considered related to romiplostim.

During the treatment period, the median (range) number of doses of investigational product was 16 (6–16) in the placebo group, 16 (4–16) in the romiplostim 500 μg group, and 16 (10–16) in the romiplostim 750 μg group. The median (range) average romiplostim dose was 391 (31–500) μg in the 500 μg group and 622 (47–750) μg in the 750 μg group. The median (range) duration of lenalidomide exposure was 19 (6–20) weeks in the placebo group, 18 (4–19) weeks in the romiplostim 500 μg group, and 18 (10–21) weeks in the romiplostim 750 μg group. The median (range) total cumulative doses of lenalidomide were 705 (290–1350) mg in the placebo group, 893 (160–1340) mg in the romiplostim 500 μg group, and 745 (280–1500) mg in the romiplostim 750 μg group.

### Efficacy

CSTEs were noted in 8 (67%) patients in the placebo group, 4 (29%) in the romiplostim 500 μg group (estimated difference from placebo group −38%; 95% CI, -74%, -2%), and 8 (62%) in the romiplostim 750 μg group (estimated difference from placebo group -5%; 95% CI, -43%, 32%) (Table 2). No linear dose response was observed. In all three treatment groups, the percentage of patients who experienced CSTEs trended higher among patients who had baseline platelet counts < 50 × 10^9^/L (Table 2). No CSTEs were reported in patients with del(5q).

**Table 2:** Clinical outcomes by baseline platelet count and deletion(5q) cytogenetic abnormality status

**Efficacy variables**	**Placebo**		**Romiplostim**			
			**500 μg**		**750 μg**	
	N^*,†^	**n (%)**	N^*^	**n (%)**	**N**	**n (%)**
Clinically significant thrombocytopenic event^‡^						
Overall	12	8 (67)	14	4 (29)	13	8 (62)
Baseline platelets <50 × 10^9^/L	5	5 (100)	5	2 (40)	5	5 (100)
Baseline platelets ≥50 × 10^9^/L	6	3 (50)	8	2 (25)	8	3 (38)
Del(5q) detected at baseline	1	0 (0)	3	0 (0)	2	0 (0)
Lenalidomide dose reduction or delay^§^						
Overall	12	6 (50)	14	5 (36)	13	2 (15)
Baseline platelets <50 × 10^9^/L	5	3 (60)	5	1 (20)	5	0 (0)
Baseline platelets ≥50 × 10^9^/L	6	3 (50)	8	4 (50)	8	2 (25)
Del(5q) detected at baseline	1	1 (100)	3	0 (0)	2	0 (0)
Achieved MDS treatment response^¶^						
Overall	12	1 (8)	14	2 (14)	13	3 (23)
Patients with baseline platelets <50 × 10^9^/L	5	0 (0)	5	1 (20)	5	0 (0)
Patients with baseline platelets ≥50 × 10^9^/L	6	1 (17)	8	1 (13)	8	3 (38)
Del(5q) detected at baseline	1	0 (0)	3	1 (33)	2	1 (50)
Achieved erythroid response^∥^						
Overall	6	2 (33)	7	2 (29)	8	2 (25)
Baseline platelets <50 × 10^9^/L	3	1 (33)	2	0 (0)	4	1 (25)
Baseline platelets ≥50 × 10^9^/L	3	1 (33)	5	2 (40)	4	1 (25)

^*^ Includes 1 patient who was found to be ineligible for the study after randomization and did not receive treatment.

^**†**^ Includes two patients who erroneously received one dose of romiplostim (500 μg and 750 μg, respectively) during the treatment period.

^‡^ Clinically significant thrombocytopenic events were defined as a platelet count <50 × 10^9^/L starting at from week 3 of cycle 1 of treatment or receipt of platelet transfusion at any time. Patients who were randomized but later found to be ineligible were counted as not having a clinically significant thrombocytopenic event.

^§^ Patients who were randomized but later found to be ineligible were counted as not having any lenalidomide dose reductions or delays.

^¶^ Complete or partial response based on 2006 modified IWG guideline [28]. Patients who were randomized but later found to be ineligible were counted as not having achieved an MDS treatment response.

^∥^ Erythroid response is defined based on the modified IWG criteria as hemoglobin increase by ≥1.5 g/dL or relevant reduction in units of red blood cell transfusions by an absolute number of ≥4 red blood cell transfusions/8 weeks.

Overall, reductions or delays in lenalidomide dosing due to thrombocytopenia were reported in 6 (50%) patients in the placebo group, 5 (36%) in the romiplostim 500 μg group, and 2 (15%) in the romiplostim 750 μg group (Table 2). In the two romiplostim groups, lenalidomide doses were reduced or delayed in a greater proportion of patients with baseline platelet counts ≥ 50 × 10^9^/L than in patients with lower baseline platelet counts; none of these patients with dose reductions/delays had del(5q) at baseline.

An overall MDS treatment response was reported in 1 (8%) patient in the placebo group, 2 (14%) in the romiplostim 500 μg group, and 3 (23%) in the 750 μg group (Table 2). Erythroid response was achieved in similar percentages of patients in the three treatment groups (Table 2).

**Figure 2:**
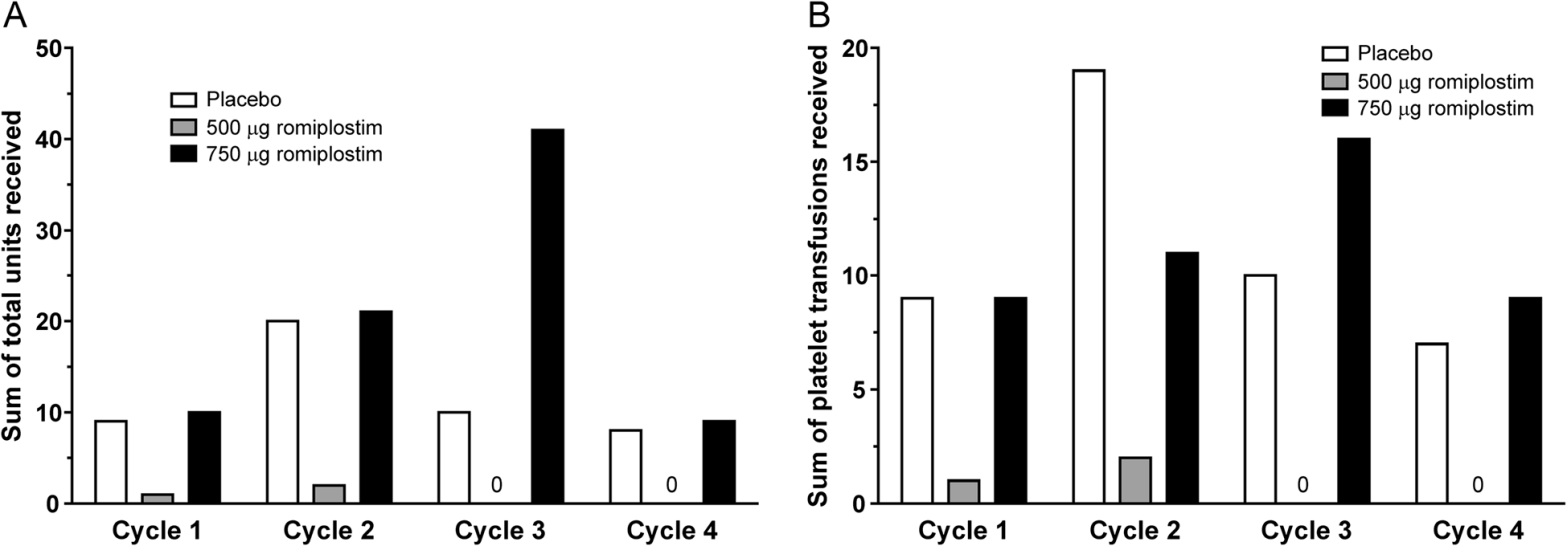
**Platelet transfusions administered. A)** Total platelet units received and **B)** total number of platelet transfusions administered.

Overall, the percentages of patients who received platelet transfusions were similar in the three treatment groups: 33% in the placebo, 29% in the romiplostim 500 μg, and 31% in the romiplostim 750 μg group. Assessment by treatment cycle showed that the percentages of patients who received transfusions in the romiplostim groups trended lower than in the placebo group during all four cycles. None of the patients in the 500 μg group received a platelet transfusion after the second cycle of treatment. In the romiplostim 500 μg group, the total number of platelet transfusions administered and the total platelet units transfused trended lower than in the placebo group during each cycle (Figure 2). In the 750 μg group, the total number of platelet transfusions administered and the total units received were generally similar to those in the placebo group (Figure 2).

**Figure 3:**
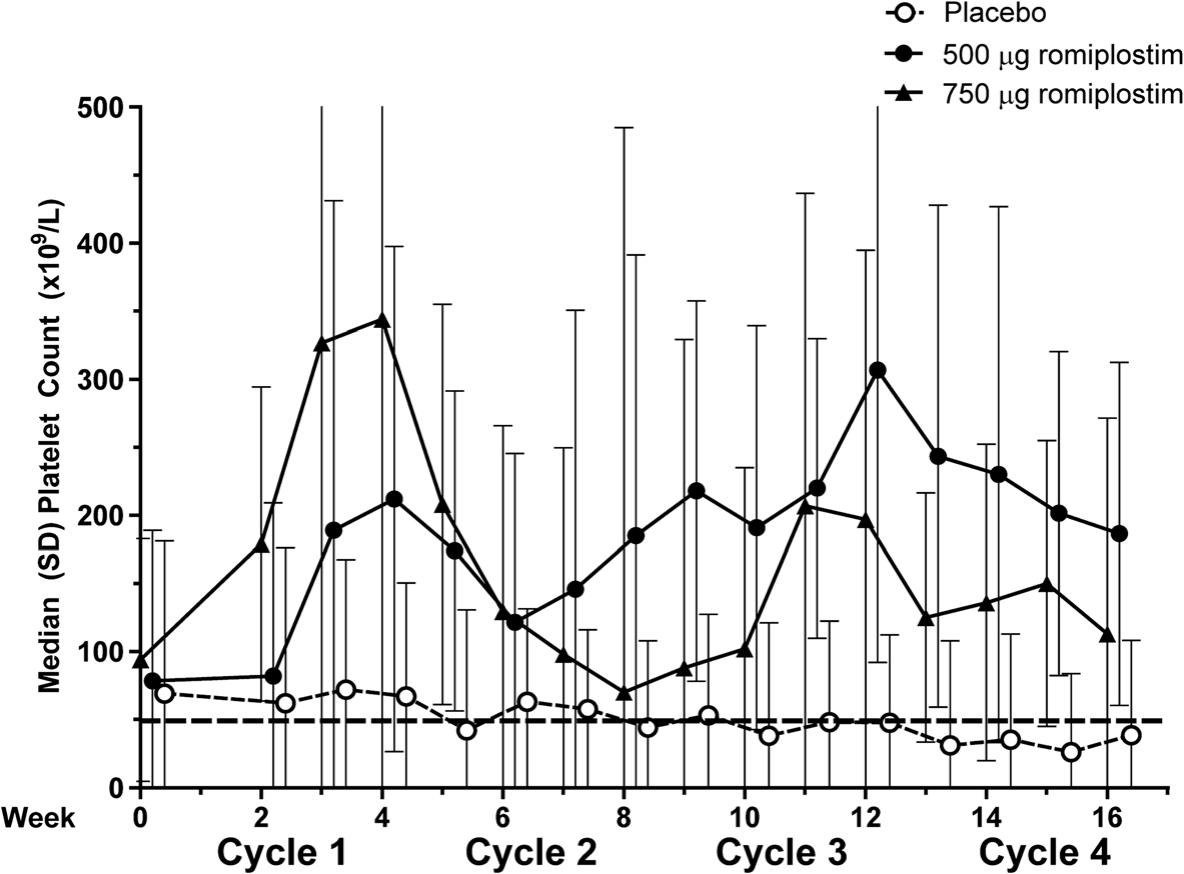
**Median platelet counts during the treatment period.** Bars of line graph represent standard deviations. Broken horizontal line is at 50 × 10^9^/L.

Median platelet counts were consistently higher in the romiplostim groups than in the placebo group throughout the treatment period (Figure 3). In the placebo group, median platelet counts generally fluctuated around 50 × 10^9^/L through week 12 and then remained below 50 × 10^9^/L. In the romiplostim 500 μg group, after the first 2 weeks, median platelet counts generally fluctuated between 150 and 250 × 10^9^/L. In the romiplostim 750 μg group, the median platelet count increased to 344 × 10^9^/L after 4 weeks and then fluctuated between 70 and 200 × 10^9^/L.

**Figure 4:**
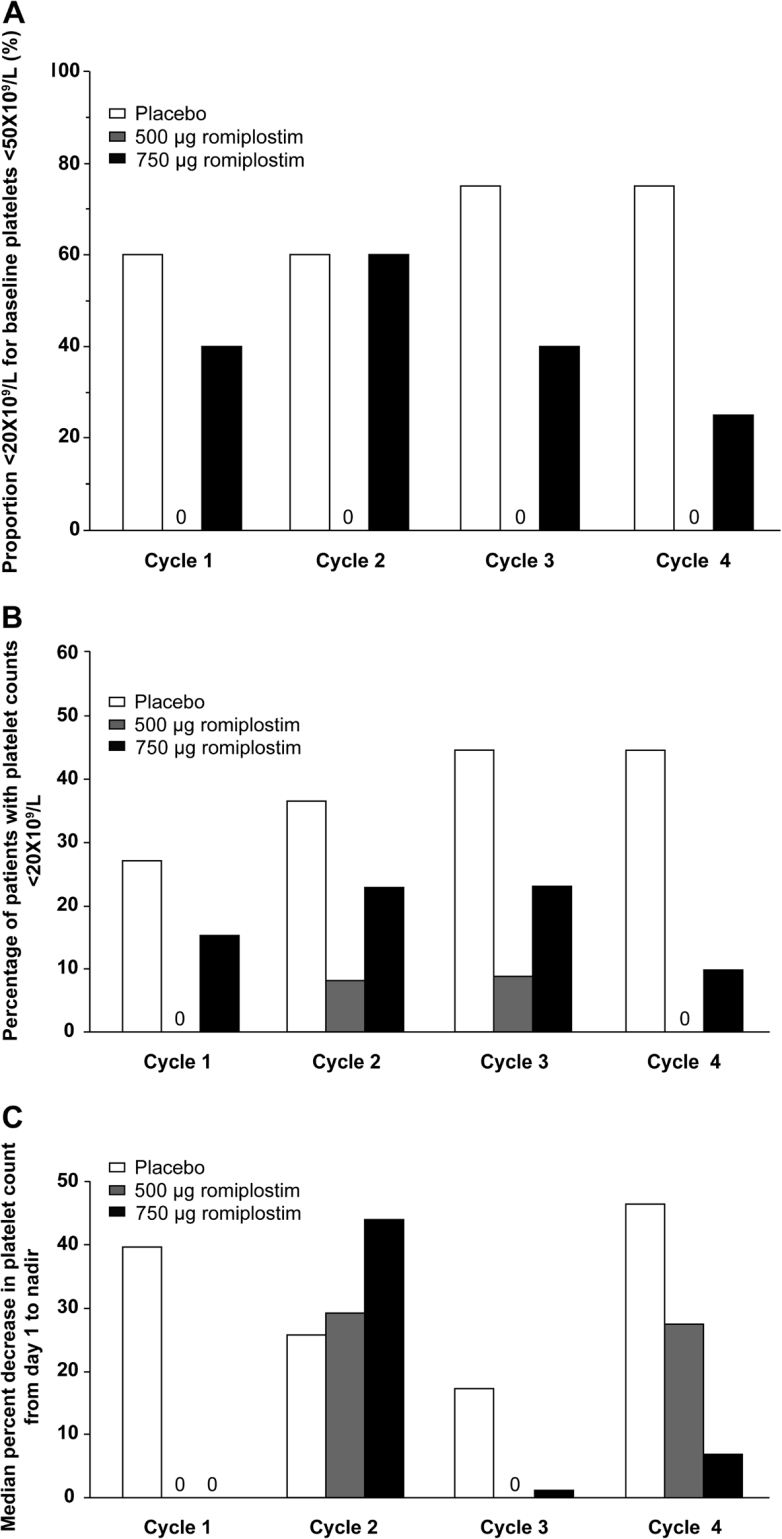
**Changes in platelet counts over treatment. A)** Proportion of patients with baseline platelet count <50 × 10^9^/L whose platelet counts were <20 × 10^9^/L at any point during the treatment cycle or overall. **B)** Percentages of patients with platelet counts <20 × 10^9^/L at any point during the treatment cycle. **C)** Median percent decrease from the platelet count at day 1 to the nadir of each treatment cycle for all patients.

Among patients whose baseline platelet counts were < 50 × 10^9^/L, none of the patients in the romiplostim 500 μg group had platelet counts < 20 × 10^9^/L during the treatment period, and the percentages of patients in the romiplostim 750 μg group with platelet counts < 20 × 10^9^/L during the treatment period trended lower than in the placebo group except during cycle 2 (Figure 4A). No patients with baseline platelet counts ≥ 50 × 10^9^/L had a drop in platelet counts to under 20 × 10^9^/L during cycle 1. During cycles 2 through 4, the percentages of patients with platelet counts < 20 × 10^9^/L in the romiplostim groups trended lower than in the placebo group and remained under 15%. Overall, the percentages of patients with platelet counts < 20 × 10^9^/L trended lower in both romiplostim groups than in the placebo group during each treatment cycle (Figure 4B).

**Figure 5:**
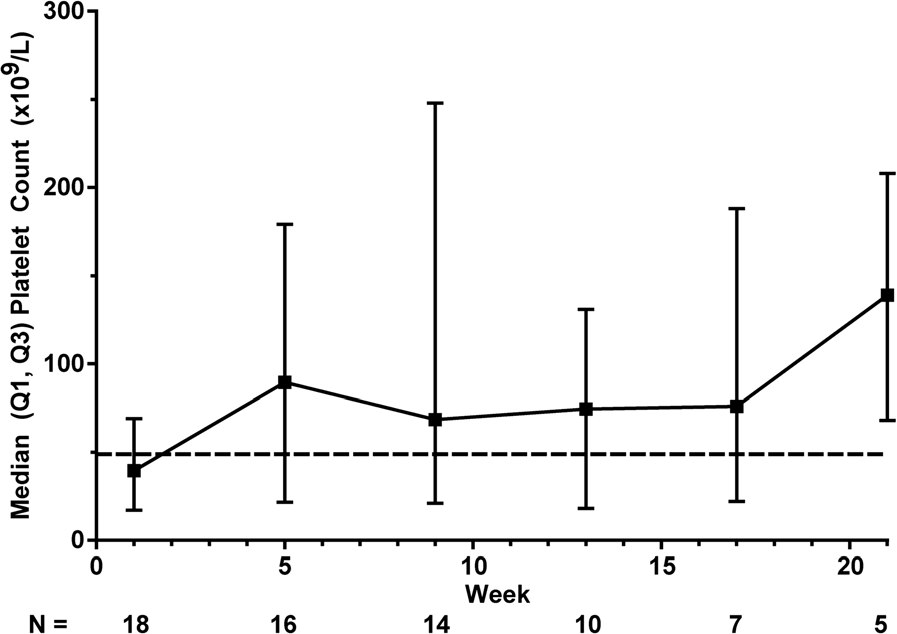
**Median platelet counts during the extension period.** Bars represent interquartile ranges. Broken horizontal line is at 50 × 10^9^/L.

The median percent decrease from the platelet count at day 1 to the nadir of each cycle is shown for all patients in Figure 4C. In the romiplostim 500 μg group, these decreases trended smaller than in the placebo group during all cycles but cycle 2 in patients with baseline platelet counts < 50 × 10^9^/L and during all cycles in patients with baseline platelet counts ≥ 50 × 10^9^/L. In the romiplostim 750 μg group, the median decreases trended smaller than those in the placebo group during all cycles except for cycle 2. During the extension period, median platelet counts generally fluctuated between 50 and 100 × 10^9^/L (Figure 5).

Bleeding events were reported in one patient in the placebo group, four in the romiplostim 500 μg group, and four in the 750 μg group. The number (95% CI) of bleeding events reported per 100 patient-weeks was 4.8 (2.1, 9.6) in the placebo group, 2.6 (0.8, 6.1) in the romiplostim 500 μg group, and 8.1 (4.7, 13.0) in the romiplostim 750 μg group.

### Safety

During the treatment period, adverse events were reported by all but one patient in the placebo group and by all patients in the romiplostim groups (Table 3). The most frequently reported adverse events were diarrhea, thrombocytopenia, neutropenia, and dizziness in the placebo group; thrombocytopenia, diarrhea, rash, and constipation in the romiplostim 500 μg group; and fatigue, diarrhea, rash, and nausea in the romiplostim 750 μg group. Adverse events led to study withdrawal or investigational product discontinuation in 3 (33%) patients in the placebo group, 2 (14%) in the romiplostim 500 μg group, and 1 (7%) in the romiplostim 750 μg group. These adverse events were pancytopenia, asthenia, and cerebrovascular accident in the placebo group; pancytopenia and rash in the romiplostim 500 μg group; and thrombocytopenia in the romiplostim 750 μg group. Only cerebrovascular accident in the placebo group was considered related to investigational product. Adverse events of severity grade ≥ 3 were reported in 6 (67%) patients in the placebo group, 9 (64%) in the romiplostim 500 μg group, and 10 (71%) in the romiplostim 750 μg group. Serious adverse events were reported in 6 (67%) patients in the placebo group, 5 (36%) in the romiplostim 500 μg group, and 4 (29%) in the romiplostim 750 μg group. Only two serious events, cerebrovascular accident in one patient in the placebo group and worsening thrombocytopenia in one patient in the romiplostim 500 μg group, were considered related to treatment. In the latter patient, worsening thrombocytopenia (platelet count of 10 × 10^9^/L) was noted at the patient’s end-of-treatment visit, 4 weeks after her last dose of romiplostim during the treatment period. When romiplostim treatment was resumed during the extension period, her platelet counts increased from 17 × 10^9^/L at extension week 1 to 86 × 10^9^/L at extension week 16, when romiplostim was discontinued because of an administrative decision.

**Table 3:** Number (%) of patients who reported adverse events during the treatment period

**n (%)**	**Placebo**	**Romiplostim**	
		**500 μg**	**750 μg**
	(N = 9)^*^	**(N = 14)**^**†**^	**(N = 14)**^**‡**^
Any adverse event (AE) ^§^	8 (89)	14 (100)	14 (100)
Grade 3	5 (56)	5 (36)	8 (57)
Grade 4	1 (11)	4 (29)	2 (14)
Most frequently reported AEs			
Fatigue	1 (11)	4 (29)	8 (57)
Thrombocytopenia	3 (33)	7 (50)	3 (21)
Diarrhea	5 (56)	5 (36)	5 (36)
Rash	2 (22)	5 (36)	5 (36)
Nausea	0 (0)	4 (29)	5 (36)
IP-related adverse events	2 (22)	7 (50)	5 (36)
Serious adverse events	6 (67)	5 (36)	4 (29)
Most frequently reported SAEs			
Thrombocytopenia	0 (0)	3 (21)	1 (7)
Anemia	0 (0)	1 (7)	2 (14)
Back pain	0 (0)	2 (14)	0 (0)
Febrile neutropenia	0 (0)	1 (7)	1 (7)
Hyperkalemia	0 (0)	1 (7)	1 (7)
IP-related SAE	1 (11)	1 (7)	0 (0)
Cerebrovascular accident	1 (11)	0 (0)	0 (0)
Worsening thrombocytopenia^¶^	0 (0)	1 (7)	0 (0)
Deaths	0 (0)	0 (0)	0 (0)
AE leading to study withdrawal or IP discontinuation	3 (33)	2 (14)	1 (7)

Legend: *AE* adverse event, *SAE* serious adverse event, *IP* investigational product.

^*^ The placebo group of the safety analysis set included the nine patients who received only placebo during the treatment period.

^**†**^ The romiplostim 500 μg group of the safety analysis set included the 13 patients treated with romiplostim 500 μg during the treatment period plus one patient originally randomized to the placebo group who inadvertently received one dose of romiplostim 500 μg during the treatment period.

^‡^ The romiplostim 750 μg group of the safety analysis set included the 13 patients treated with romiplostim 750 μg during the treatment period plus one patient originally randomized to the placebo group who inadvertently received one dose of romiplostim 750 μg during the treatment period.

^§^ No Grade 5 adverse events were reported.

^¶^ This event was reported 4 weeks after the patient’ s last dose of romplostim during the treatment period.

During the extension period, the safety profiles of the investigational products were similar to those during the treatment period. Serious adverse events were reported in 4 (22%) patients. Of these events, only leukocytosis, reported in one patient who had received placebo during the treatment period, was considered related to investigational product. One patient in the romiplostim 750 μg group died during the extension period from an intestinal obstruction, which was not considered related to investigational product. No patients developed neutralizing antibodies to romiplostim or thrombopoietin. For the 37 patients who had available screening and end-of-treatment bone marrow biopsy data, no patients exhibited changes in trichrome stain indicative of collagen fibrosis formation. No clinically meaningful differences were detected in bone marrow reticulin/collagen formation between placebo and romiplostim treatment groups.

No patients met the study-defined criteria for disease progression to AML (i.e., marrow or peripheral blast cell count ≥ 20% with confirmation 4 weeks after withdrawal of romiplostim) during the treatment or extension period. Two patients, one in the romiplostim 500 μg group and the second in the romiplostim 750 μg group, were reported as having an increase in bone marrow blasts to greater than 20%, consistent with the WHO definition of AML, but both were receiving romiplostim at the time of the blast increase. The patient in the 500 μg group at study entry was a 63-year-old woman with an IPSS score of 1.5 (whose entry into the trial was due to a protocol violation), a WHO classification of RAEB-1, and a bone marrow blast count of 5%. Her prognostic score, based on the paper by Garcia-Manero et al., was 5 (category 3: median survival, 14.2 months; 4-year survival, 7%) [8]. At 2 months, her bone marrow blast count had increased to 24%. Because of a concern for possible disease progression, she withdrew her consent and discontinued the study. She declined to undergo a follow-up bone marrow biopsy. She died 1 year later. The second patient in the 750 μg group at study entry was a 69-year-old woman with an IPSS score of 1.0, a WHO classification of RAEB-1, and a bone marrow blast count of 5%. Her prognostic score was 6 (category 3: median survival, 14.2 months; 4-year survival, 7%) [8]. Four days after her last dose of romiplostim at week 16, her blast count had increased to 29%. She discontinued the study 3.5 weeks later because of the increased blast count. The patient refused to undergo an additional bone marrow biopsy. No additional follow-up information is available.

## Discussion

Analysis of the results of this study suggests that romiplostim can reduce the rate of CSTEs while increasing platelet counts in lower risk MDS patients receiving lenalidomide. These effects were reflected in trends toward smaller percentages of patients requiring lenalidomide dose reductions or delays, higher percentages of patients achieving MDS treatment response, and lower percentages of patients receiving transfusions during each lenalidomide cycle in the romiplostim groups than in the placebo group. There was no observed romiplostim dose response identified, possibly because of the small numbers of patients in each treatment group.

The results of this study were consistent with those of two previous studies in which MDS patients received romiplostim in combination with the DNA methyltransferase inhibitors azacitidine or decitabine [23,25]. In those studies, trends toward lower platelet transfusion rates, higher platelet counts at the beginning and nadir of each treatment cycle, and lower percentages of patients with bleeding events in the romiplostim-treated groups were observed. These results suggested that adding romiplostim to methyltransferase inhibitor therapy was associated with clinical benefits. Attempts to compare our results with those of studies of romiplostim monotherapy in MDS patients were confounded by differences in study design, severity of disease in the patients studied, and the outcome variables assessed [19,20].

In this study, the overall MDS response to lenalidomide appeared to be modestly affected by romiplostim. In previous reports, thrombocytopenia was shown to be common among MDS patients receiving lenalidomide and is postulated to constitute a predictive indicator of therapeutic response [16], as well as a frequent cause of lenalidomide dose reduction and interruption [13-15]. In one study of lenalidomide in del(5q) MDS patients [13], the duration of lenalidomide treatment was significantly less in patients with baseline thrombocytopenia because of recurring dosing interruptions due to myelosuppression. In that study, thrombocytopenia was a significant predictor of a lower probability of transfusion independence. These findings suggest that duration of lenalidomide treatment is an important determinant of hematologic improvement. In other studies, severe thrombocytopenia in MDS patients treated with lenalidomide generally occurred within the first two cycles, with median times to dose adjustments ranging from 22 to 43 days [13-15]. Here in our study, romiplostim treatment was associated with increases in platelet counts by the third week of treatment, and lenalidomide dose reductions or delays tended to be lower in patients treated with romiplostim. These effects may have contributed to the observed higher rates of MDS responses in the romiplostim- versus placebo-treated groups.

Adverse events in this study were similar in the three treatment groups, with only two serious events (cerebrovascular accident, worsening thrombocytopenia) considered treatment related. No neutralizing antibodies to romiplostim were detected in any patients, and there was no evidence of increased bone marrow reticulin or collagen formation in individuals treated with drug. Although possible transformation to AML was noted in two patients, these patients did not meet protocol-defined criteria for AML because of the lack of repeat marrow evaluation after a 4-week drug washout period. Moreover, the reported increases in marrow blasts were noted in both patients while they were still receiving romiplostim, which may transiently increase blast cell counts [18]. Therefore, it remains unclear whether these cases represent transient drug-related blast increases or true AML progression. It is noteworthy that the data monitoring committee recommended discontinuing investigational treatment in a previous randomized trial of romiplostim in MDS patients [22]. At the time of investigational treatment discontinuation in that trial, a non-significant numeric imbalance of AML cases was observed; there were concerns that the potential small benefit seen in the reduction of bleeding did not outweigh the potential risk for disease progression to AML and that transient increases in blast cell counts may put patients at risk for diagnosis of and treatment for AML. As a result of these events, a second trial was modified by Amgen administratively such that patients discontinued romiplostim and entered long-term follow-up [21].

In conclusion**,** the results of this study suggest that romiplostim may reduce the rate of CSTEs while increasing platelet counts in lower risk MDS patients receiving lenalidomide. Our data show that romiplostim can decrease the frequency of lenalidomide dose reductions and delays due to thrombocytopenia and is potentially associated with improved treatment responses. Inferences drawn from this study are limited by the small sample size, enrollment of patients with variable degrees of baseline thrombocytopenia, and imbalances in MDS disease characteristics between treatment groups. Additional study is needed to confirm the results of this preliminary trial.

## Abbreviations

AE: Adverse event; AML: Acute myeloid leukemia; CI: Confidence interval; CR: Complete response; CSTE: Clinically significant thrombocytopenic event; Del(5q): Interstitial deletion of the long arm of chromosome 5; ECOG: Eastern Cooperative Oncology; eTPO: Endogenous thrombopoietin; FDA: Food and Drug Administration; GCP: Good Clinical Practice; ICH: International Conference on Harmonisation; IP: Investigational product; IPSS: International Prognostic Scoring System; ITP: Immune thrombocytopenia; IVRS: Interactive voice response system; IWG: International Working Group; MDS: Myelodysplastic syndrome; MDS-U: Myelodysplastic syndrome, unclassified; PR: Partial response; Q1: 25^th^ percentile; Q3: 75^th^ percentile; RA: Refractory anemia; RAEB: Refractory anemia with excess blasts; RARS: Refractory anemia with ringed sideroblasts; RCMD: Refractory cytopenia with multilineage dysplasia; RCMD-RS: Refractory cytopenia with multilineage dysplasia and ringed sideroblasts; SAE: Serious adverse event; SD: Standard deviation; TI: Transfusion independence; WHO: World Health Organization.

## Competing interests

ESW served on an advisory board for Amgen Inc. in 2011 and received a consulting fee. RML has served as a consultant and on a speakers’ bureau for Amgen Inc. and has received honoraria and research funding from Amgen Inc. RAL has received research support from Amgen. SG, DL and CM declare that they have no competing interests. BS has served as a speaker and consultant for Celgene. KH and AY are employees and stockholders of Amgen, Inc.

## Authors' contributions

All authors were involved with data acquisition, reviewed and revised the manuscript for important intellectual content, and gave final approval of the version to be published. In addition, KH contributed to the conception and design of the study, ESW was involved in the drafting of the manuscript, and KH and AY analyzed and interpreted the data.
